# Burnout and Related Factors of Nurses Caring for DNR Patients in Intensive Care Units, South Korea

**DOI:** 10.3390/ijerph17238899

**Published:** 2020-11-30

**Authors:** Sohyune Sok, Hyebeen Sim, Bokhee Han, Se Joung Park

**Affiliations:** 1 College of Nursing Science, Kyung Hee University, Seoul 02447, Korea; 2 Department of Nursing, Graduate School, Kyung Hee University, Seoul 02447, Korea; simsixty@naver.com (H.S.); angelbokhee@khu.ac.kr (B.H.); sdams3004@khu.ac.kr (S.J.P.)

**Keywords:** ICU, DNR, burnout

## Abstract

This study examined the factors related to burnout, depression, job stress, and job satisfaction in intensive care unit (ICU) nurses caring for do not resuscitate (DNR) patients, as well as analyzed any differences. A cross-sectional descriptive design was employed. Study participants involved a total of 115 nurses caring for DNR patients in ICUs in South Korean hospitals. Measures involved a demographic form, Copenhagen Burnout Inventory (CBI), Center for Epidemiologic Studies Depression Scale, Nursing Job Stress Scale (Korean version), and Nursing Job Satisfaction Scale (Korean version). Data were collected from February to March 2017. The analyses illustrated a higher level of burnout, a slightly lower level of depression, a slightly lower level of nursing job stress, and a very slightly higher level of nursing job satisfaction compared with the median value of the score range for each scale. Burnout had a significant, positive relationship with depression and nursing job stress, and depression had a significant, positive relationship with nursing job stress. This study illuminates preliminary evidence that ICU nurses who are caring for DNR patients have a higher level of burnout compared with the median value of the score range in the CBI (Korean version). Burnout, depression, job stress, and job satisfaction were interrelated in ICU nurses.

## 1. Introduction

In modern society, the average human life expectancy has been extended due to the development of medical technology and the mortality rate has decreased [1,2]. In addition, the number of elderly with geriatric diseases has increased [2,3]. This prolonging of human life has been possible due to an increasing number of diseases which were previously difficult to treat now being successfully treated [4,5]. However, this has also caused problems due to the meaningless life extension and resuscitation of patients who are difficult to revive [6].

In South Korea, the incidence rate of do not resuscitate (DNR) decisions is 73.5% among intensive care unit (ICU) patients, of which 68% are older than 65 years [3]. Once admitted to the ICU, patients and their family members are confused by the explanations provided by medical staff regarding the patient’s condition [7,8,9,10]. It is difficult for the medical staff and caregivers to agree on a treatment goal, which might cause pain to the family members until the patient dies [11,12,13,14,15]. Life-sustaining treatment may deprive the opportunity of other critically ill patients to use the beds and waste unnecessary medical resources, thereby resulting in problems such as inefficient distribution of medical resources [16,17]. With regard to such life-sustaining treatment to extend human life, nurses with field experience may regret their decisions and conflict with reality, whereby they cannot reflect their opinions as professionals to the family and patient regarding their decision-making with respect to DNR [18,19]. They also experience negative feelings, such as guilt and sorrow, which are accompanied by depression, job stress, and burnout [11,20,21].

Nurses in ICUs caring for dying patients suffering from negative psychological reactions (e.g., fear of death, isolation, loneliness, dread, and anxiety) suffer from stress unlike that faced by nurses in other departments caring for general patients [20,21,22,23]. This is because they lack diverse and specialized knowledge related to hospice end-of-life care and the use of end-of-life nursing guidelines for care and preparation for dying, despite their practice field [10,22,23]. Moreover, it is difficult to provide nursing in terms of emotional, psychological, and spiritual aspects due to a lack of enough time to provide proper hospice care for DNR patients [9,14,24]. When anticipating the death of a DNR patient and watching the separation process for family and society, leading to anxiety and pain, highly skilled nursing is necessary in order to successfully provide care for the various psychological and social difficulties experienced by dying patients and their family members, as well as the extreme pain, anxiety, and uncontrollable anger expressed by the dying patients [18,21,22,25]. In addition, ICU nurses are less likely to feel tense when taking care of DNR patients compared to the other patients, with feelings of regret and guilt associated with their reduced level of care. As a result, they suffer from serious physical and psychological difficulties [6,18]. Because of this, research is needed that focuses on nurses caring for DNR patients in ICUs and not in other departments.

Most previous studies related to DNR patients were qualitative studies on the perceptions and experiences of doctors and nurses [10,11,13,18], ethical attitudes [2,4,6], changes in nursing activities [13,14,16,20], and perceptions and sorrow of patients and their families [17,26]. However, studies conducted on burnout, depression, job stress, and job satisfaction of the nurses caring for an increasing number of DNR patients are insufficient. Therefore, this study was conducted to measure the factors related to burnout, depression, job stress, and job satisfaction of ICU nurses caring for an increasing number of DNR patients, as well as to analyze any differences, so as to provide basic data on quality nursing practice for DNR patients and their families. The aims of this study were (1) to identify the general characteristics of study participants, (2) to examine their levels of burnout, depression, job stress, and job satisfaction, (3) to examine the correlations among study variables, and (4) to examine differences in burnout, depression, job stress, and job satisfaction according to the general characteristics of study participants. 

## 2. Material and Methods

### 2.1. Study Design and Participants

A cross-sectional descriptive design was adopted. The study participants involved a total of 115 nurses caring for DNR patients in three hospitals in Seoul, South Korea. These three hospitals were very similar in size and organization, number of beds, and number of medical personnel and staff. They were recruited through convenient sampling. The eligibility criteria included nurses aged 20 years or older who were working in intensive care units and who agreed to take part in the study, with full capability to verbally understand Korean. A total of 116 (96.7%) of the 120 questionnaires were returned. Due to incomplete data, only 115 questionnaires were included in the final dataset. The sample size adequacy (*n* = 109) was found to be adequate using a correlation statistical test in G power 3 analysis with an alpha level of 0.05, medium effect size of 0.30, and power of 0.90 [27].

### 2.2. Measures

The study participants’ general characteristics consisted of gender, age, marital status, educational level, current ward, salary level, duty shift, total working experience, current career experience, and motivation to become a nurse.

The Copenhagen Burnout Inventory (CBI) was developed by Kristen et al. [28] and translated to the Korean version by Him [29]. This scale was used to measure the level of burnout of the participants. It consisted of a total of 19 questions using a five-point Likert scale with three subcategories (personal burnout, job related burnout, and patient-related burnout). The range of scores was 19–95, with higher scores indicating a higher level of burnout. The reliability of this instrument was deemed good with Cronbach’s α = 0.94.

The Center for Epidemiologic Studies Depressed Mood Scale (CES-D) was developed by the National Institute of Mental Health’s Center for Epidemiologic Studies, United States of America (USA) [30]. Chon et al. [31] developed an integrated Korean version of the CES-D, which was used to measure the level of depression of the participants, consisting of a total of 15 yes (one point) and no (zero points) questions. The range of scores was 0–15 scores, with higher scores indicating a higher level of depression. The reliability of this instrument was deemed good with Cronbach’s α = 0.89.

The Nursing Job Stress Scale was developed by Hingley [32] and translated to the Korean version by Kim [33]. This scale was used to measure the level of nursing job stress of the participants. It consisted of a total of 30 questions using a five-point Likert scale. The range of scores was 30–150, with higher scores indicating a higher level of nursing job stress. The reliability of this instrument was deemed good with Cronbach’s α = 0.93.

The Nursing Job Satisfaction Scale was developed by Paula [34] and translated to the Korean version by Han and Mun [35]. This scale was used to measure the level of nursing job satisfaction of the participants. It consisted of a total of 20 questions using a five-point Likert scale. The range of scores was 20–100, with higher scores indicating a higher level of nursing job satisfaction. The reliability of this instrument was deemed good with Cronbach’s α = 0.89.

### 2.3. Procedure

The duration of data collection was from February to March 2017. Three hospitals with ICUs were visited to obtain permission for this study. The researchers contacted the prospective study participants and discussed with them the purpose of this study, the details of participation, and the questionnaire to be used for data collection. The authors received written informed consent from several ICU nurses who volunteered to provide valuable information for this study. All five questionnaires were given only to nurses who provided consent, all of which comprised self-reporting questionnaires administered by the researchers. The average time taken to fill out the questionnaires was 20–25 min.

### 2.4. Statistical Analysis

The collected data were analyzed using the SPSS version 21.0 statistical software program. The general characteristics of the study participants and the levels of study variables were analyzed using descriptive statistics (frequency, percent, mean, and standard deviation). Correlations between study variables were analyzed using Pearson’s correlation coefficient. A *t*-test and ANOVA were used to determine the level of differences in study variables according to the general characteristics of the study participants, while Scheffe’s method was used as a post hoc test. A *p*-value less than 0.05 was considered statistically significant.

### 2.5. Ethical Considerations

This study was approved by the Institutional Review Board of Kyung Hee University, Seoul, South Korea (KHSIRB-17-004). Study participants were informed about the purpose, methods, and procedures of the study; they were also told that their participation was voluntary, and they had the right to withdraw from the study at any time. Participants were also informed about the anonymity of the data obtained from the study. Researchers received completed written consent forms from those who agreed to participate in the study.

## 3. Results

### 3.1. General Characteristics of Study Participants

Women (92.2%) represented the primary gender in the study group. The average age was 29.75 years, with 55.7% of participants in the 25–30 year old bracket. Moreover, 84.3% of participants were single, and 73.0% graduated university. Furthermore, 71.3% of participants were working in medical intensive care unit (MICUs) with a maximum of three shifts. Total working experience was on average 4.50 years, with most (66.1%) participants having less than 5 years of experience. Guaranteed employment after graduation was the most common source of motivation to become a nurse (42.6%) (Table 1).

### 3.2. Levels of Burnout, Depression, Job Stress, and Job Satisfaction

The mean score of participants on the Korean version of the CBI was 64.03 (*SD* = 11.64), indicating a higher level of burnout compared to the median value (57 points) of score range (19–95). The mean score on the Korean version of the CES-D was 6.10 (*SD* = 4.01), showing a slightly lower level of depression compared to the median value (7.5 points) of the score range (0–15). The mean score on the on the Korean version of the Nursing Job Stress Scale was 86.23 (*SD* = 16.11), exhibiting a slightly lower level of job stress compared to the median value (90 points) of the score range (30–150). The mean score on the Korean version of the Nursing Job Satisfaction Scale was 62.87 (*SD* = 21.01), demonstrating a very slightly higher level of job satisfaction compared to the median value (60 points) of the score range (20–100).

### 3.3. Correlations among Study Variables

Burnout had a significant, positive relationship with depression (*r* = 0.47, *p* < 0.001) and job stress (*r* = 0.57, *p* < 0.001), but a significant, negative relationship with age (*r* = −0.20, *p =* 0.032). Depression had a significant, positive relationship with job stress (*r* = 0.19, *p* = 0.038), but a significant, negative relationship with job satisfaction (*r* = −0.22, *p* < 0.018), age (*r* = −0.37, *p* < 0.001), total working experience (*r* = −0.33, *p* < 0.001), and current career experience (*r* = −0.37, *p* < 0.001) (Table 2).

### 3.4. Differences in Burnout, Depression, Job Stress, and Job Satisfaction according to General Characteristics of Study Participants

In burnout, there were significant differences according to gender (*t* = 2.607, *p* = 0.028, *df* = 114), whereby women scored higher than men. In depression, there were significant differences according to age (*F* = −3.866, *p* = 0.006, *df* = 114), marital status (*t* = −2.157, *p* = 0.042, *df* = 114), education level (*F* = 3.331, *p* = 0.039, *df* = 114), salary level (*F* = 5.646, *p* = 0.001, *df* = 114), total working experience (*F* = 4.930, *p* = 0.009, *df* = 114), and current career experience (*F* = 6.822, *p* = 0.002, *df* = 114). In terms of depression, participants under the age of 25 scored higher than those over 40, while single participants scored higher than married people and participants who graduated from college scored higher than those who completed graduate school. Participants who earned a monthly salary of 300 million KRW or less scored higher than those who earned more than this threshold. Participants with both a total working experience and a current career experience of less than 5 years scored higher than those with more than 10 years of experience. In job stress, there was a significant difference according to education level (*F* = 5.825, *p* = 0.004, *df* = 114), whereby those who completed graduate school scored higher than university graduates (Table 3). 

## 4. Discussion

In comparison with other studies using the same tool, the average score of 3.37 established in this study was higher than the 2.88 found by Him [29], who conducted path analysis on emotional labor and burnout of nurses, and the 3.08 found by Yoon and Kim [36]. This result suggests that the burnout degree of ICU nurses in the current study was higher due to the increased tension and conflict as a result of their special work situation, in addition to a greater psychological and mental burden stemming from their care of DNR patients [8,29].

Our findings suggest greater depression in ICU caring for DNR patients than those caring for general patients, as a result of the guilt and sadness linked to a perceived lack of end-of-life care [17,18]. These emotions also lessened with experience [10].

A lower level of job stress was found with respect to the study by Ji and Yoo [23] for nurses in general wards and ICUs. It would appear that job stress is related to the severity of the patients’ illness, leading to a heavy workload in medical institutions. However, ICU nurses are considered to face further stress due to the burden of simultaneous end-of-life care for acute and DNR patients [6,7].

The job satisfaction of ICU nurses was also found to be low due to this heavier workload. In a previous study conducted by Kim and Lee [37] on general ward and ICU nurses, job satisfaction of the latter was lower than that of the former. In a study by Yang [38] on nurses from various wards, the job satisfaction of ICU nurses was significantly lower than that of the nurses in surgical and medical wards, in contrast to the results of this study. ICU nurses are commonly exposed to urgent situations where they may experience various risk factors when caring for patients in a persistently unstable state. However, in this study, the job satisfaction of ICU nurses was very slightly higher than the median value. The benefits provided by the hospital organization to appropriately compensate for the increased risk may have contributed to the improved satisfaction level of ICU nurses, whereby intrinsic satisfaction (e.g., utilization of abilities, sense of achievement, employment stability, development possibility, social status, and responsibility) is considered high; therefore, further research on this is deemed necessary.

In the correlation analysis, burnout, depression, and job stress were positively correlated in ICU nurses caring for DNR patients, thereby showing consistent results with those of other studies. Dedication is very important in the therapeutic relationship between the nurse and patient. However, when nurses become tired and the risk of burnout increases, the levels of depression and job stress also increase, thereby lowering job satisfaction, which may ultimately increase turnover intention.

On the other hand, depression was significantly higher in participants below 25 years old than in those aged 40 years or older, thereby showing a difference according to age. Depression was also significantly higher in unmarried subjects than in married ones, thereby indicating that marital status also affects the degree of depression.

This result is attributable to the fact that younger nurses generally have less clinical experience, a more insufficient work proficiency, and a higher psychological and mental burden when taking care of DNR patients, whereby they cautiously respond to the changing hospital environment. In addition, married people are typically older than unmarried people, with potentially greater career stability and increased clinical experience, placing them in a work position where their opinions care more reflected. Therefore, the degree of depression may have been lower in married people due to the difference in knowledge and proficiency among nurses. Job stress was found to be statistically significantly higher when the final education level was higher, in line with the occupational social status of subjects with more than a master’s degree being higher with a consequent increase in workload. 

According to the results of this study, nursing interventions that reduce the burnout of ICU nurses taking care of DNR patients are deemed necessary. In particular, strategies or nursing interventions aimed at lowering the depression level of new ICU nurses below 25 years of age are required. The results of this study can be used as supporting data when applying interventions for ICU nurses. This study also contributes to an enhancement of the literature on related research. In the future, repetitive studies, including the same variables, are necessary while expanding the sample size of ICU nurses caring for DNR patients. In order to better understand the inner world of ICU nurses with regard to burnout, it will be necessary to conduct a qualitative research. In addition, experiments should be conducted in order to verify the effect of interventions on lowering the burnout degree. 

The possibility for generalization of the results of this study is limited. As the study participants were only recruited as nurses working at general hospitals in Seoul, South Korea, the characteristics of the resulting data are limited. Furthermore, the small sample size may be a further limitation leading to potential false negatives. Therefore, it is recommended to replicate this study using larger samples from the same country and from different regions to confirm the potential generalizability of the study results.

## 5. Conclusions

In conclusion, the burnout level of ICU nurses caring for DNR patients was higher than the median value, while their burnout, depression, and job stress were positively correlated. The level of depression was highest among ICU nurses below 25 years old. According to these results, strategies or intervention methods are needed to reduce the burnout of ICU nurses caring for DNR patients.

## Figures and Tables

**Table 1:** General characteristics of the study participants.

Characteristics	*n* (%)
Gender	
Female	106 (92.2)
Male	9 (7.8)
Age (years)	
<25	5 (4.3)
25–30	64 (55.7)
30–35	29 (25.2)
35–40	10 (8.7)
≥40	7 (6.1)
Mean (SD)	29.75 (4.93)
Marital status	
Single	97 (84.3)
Married	18 (15.7)
Education level	
College	23 (20.0)
University	84 (73.0)
Graduate school	8 (7.0)
Current ward	
Medical intensive care unit (MICU)	82 (71.3)
Surgical intensive care unit (SICU)	19 (16.5)
Neuro (surgical) intensive care unit (NCU)	10 (8.7)
Neonatal intensive care unit (NICU)	4 (3.5)
Salary level (million KRW/month)	
201–250	53 (46.1)
251–300	42 (36.5)
301–350	13 (11.3)
351	7 (6.1)
Duty	
Days	5 (4.3)
Three shifts	110 (95.7)
Total working experience (years)	
<5	76 (66.1)
5–10	25 (21.7)
>10	14 (12.2)
Mean (SD)	4.50 (4.48)
Current career experience (years)	
<5	84 (73.0)
5–10	22 (19.1)
>10	9 (7.8)
Mean (SD)	3.64 (3.37)
Motivation to become a nurse	
Aptitude and interest	23 (20.0)
Family members	30 (26.1)
To help others	13 (11.3)
Guaranteed employment after graduation	49 (42.6)

**Table 2:** Correlations among study variables.

Variables	Burnout	Depression	Job Stress	Job Satisfaction	Age	Total Working Experience	Current Career Experience
Burnout	1						
Depression	0.47 *	1					
Job stress	0.57 *	0.19 *	1				
Job satisfaction	−0.11	−0.22 *	0.03	1			
Age	−0.20 *	−0.37 *	0.03	−0.01	1		
Total working Experience	−0.05	−0.33 *	0.05	0.03	0.83 *	1	
Current career experience	−0.10	−0.37 *	−0.06	0.08	0.69 *	0.61 *	1

* *p* < 0.05.

**Table 3:** Differences in burnout, depression, job stress, and job satisfaction according to general characteristics of study participants.

Characteristic	Burnout	*t*/*F*	Depression	*t*/*F*	Job Stress	*t*/*F*	Job Satisfaction	*t*/*F*
	Mean (SD)		Mean (SD)		Mean (SD)		Mean (SD)	
Gender								
Female	64.85 (11.30)	2.607 *	6.26 (4.03)	1.606	86.55 (16.17)	0.751	62.75 (21.83)	−0.507
Male	54.22 (11.78)		4.33 (3.39)		82.44 (15.70)		64.22 (5.91)	
Age (years)		1.785		3.866 *		0.976		0.143
<25	96.40 (4.56)		8.80 (3.63)		84.40 (15.02)		59.60 (3.84)	
25–30	65.15 (10.86)		6.78 (3.96)		86.00 (15.38)		64.06 (27.43)	
30–35	62.10 (12.11)		5.45 (4.08)		84.72 (16.47)		60.96 (7.34)	
35–40	66.00 (14.55)		5.50 (3.17)		95.40 (21.11)		62.00 (7.48)	
≥40	55.00 (12.80)		1.57 (0.97)		82.71 (13.81)		63.42 (9.69)	
Marital status								
Single	64.76 (11.27)	1.43	6.45 (3.92)	2.157 *	85.41 (15.53)	−1.106	62.98 (22.64)	0.257
Married	60.05 (13.09)		4.22 (4.05)		90.61 (18.77)		62.22 (8.12)	
Education level		3.268		3.331 *				0.200
College	69.39 (11.78)		7.65 (4.51)		93.17 (14.22)	5.825 *	60.39 (6.06)	
University	62.84 (11.52)		5.91 (3.86)		83.28 (15.51)		63.54 (24.31)	
Graduate school	61.00 (8.64)		3.75 (2.49)		97.13 (18.41)		63.00 (5.95)	
Current ward		0.918		0.937		3.608		0.259
MICU	64.81 (12.36)		6.28 (4.13)		88.65 (16.37)		63.21 (24.56)	
SICU	64.11 (9.56)		6.42 (3.94)		83.68 (13.69)		59.68 (5.57)	
NCU	58.80 (9.47)		5.20 (3.48)		72.10 (10.69)		63.80 (6.39)	
NICU	60.50 (9.43)		3.25 (1.71)		83.75 (16.50)		68.75 (6.89)	
Salary level		1.619		5.646 *		1.474		0.588
(million KRW/month)								
201–250	65.30 (11.39)		7.06 (4.03)		85.28 (16.50)		63.83 (30.26)	
251–300	61.83 (11.12)		6.26 (3.75)		84.40 (15.01)		59.93 (6.07)	
301–350	68.23 (13.57)		4.15 (3.55)		94.53 (17.77)		64.77 (5.11)	
351	59.71 (11.41)		1.57 (1.51)		88.85 (14.35)		69.71 (3.73)	
Duty shift		0.705		−1.580		0.990		−0.865
Days	68.00 (12.94)		4.20 (2.68)		92.60 (14.65)		59.60 (7.56)	
Three shifts	63.83 (11.61)		6.19 (4.04)		85.93 (16.17)		63.01 (21.42)	
Total working		0.461		4.930 *		1.370		2.174
experience (years)								
<5	64.05 (10.94)		6.86 (0.46)		86.01 (16.01)		60.45 (6.62)	
5–10	65.32 (11.89)		5.20 (3.86)		83.48 (14.27)		70.44 (3.02)	
>10	61.57 (15.06)		3.64 (2.95)		92.28 (19.12)		62.50 (7.41)	
Current career		2.017		6.822 *		0.769		3.180
experience (years)								
<5	64.79 (10.32)		6.86 (3.95)		87.33 (16.51)		60.27 (6.39)	
5–10	64.09 (13.90)		4.50 (3.66)		82.72 (14.55)		72.72 (5.59)	
>10	56.67 (15.86)		2.89 (2.57)		84.44 (16.14)		63.00 (8.35)	
Motivation to become a nurse		0.404		0.512		0.352		1.577
Aptitude and interest	61.74 (14.30)		5.48 (4.12)		88.95 (17.16)		71.09 (4.86)	
Family members	65.20 (10.57)		6.03 (4.15)		86.26 (16.73)		59.27 (6.96)	
To help others	64.15 (11.08)		5.53 (3.79)		86.76 (18.18)		62.31 (6.18)	
Guaranteed employment after graduation	64.34 (11.24)		6.59 (3.97)		84.77 (14.95)		61.36 (6.63)	

* p < 0.05.
